# Role of Emotional Attachment and Reciprocity in Sons’ Perceived Care Motivation for Parental Figures

**DOI:** 10.1093/geroni/igab046.1627

**Published:** 2021-12-17

**Authors:** Tomoko Wakui, Ryo Hirayama, Ichiro Kai

**Affiliations:** 1 Tokyo Metropolitan Institute of Gerontology, Tokyo, Tokyo, Japan; 2 Osaka City University, Osaka, Osaka, Japan; 3 The University of Tokyo, Tokyo, Tokyo, Japan

## Abstract

The collapse of the traditional Japanese household system and the subsequent social advancement of women has led supporting parents as a family matter, and led more men to assume caregiving roles; however, very few studies have focused on sons’ care motivation. This study aimed to understand adult sons’ perceived care motivation and to examine the respective related factors of emotional attachment and reciprocity. A total of 1322 men (M [age] = 44.5) participated in a web-based questionnaire survey. Perceived care motivation for providing five types of support (e.g., helping with daily activities and housework) to each parent and parent-in-law was assessed. Regression analyses revealed that emotional attachment with parents and parents-in-law predicted perceived care motivation for all types of support. Furthermore, the role of reciprocity was indicated by the association between rearing by mother-in-law and son-in-law’s motivation to provide assistance in financial matters, housework, and visiting a hospital.

